# Regulation of Gut Microbiota on Immune Reconstitution in Patients With Acquired Immunodeficiency Syndrome

**DOI:** 10.3389/fmicb.2020.594820

**Published:** 2020-10-27

**Authors:** Shi-Tao Geng, Zun-Yue Zhang, Yue-Xin Wang, Danfeng Lu, Juehua Yu, Jian-Bo Zhang, Yi-Qun Kuang, Kun-Hua Wang

**Affiliations:** ^1^NHC Key Laboratory of Drug Addiction Medicine, First Affiliated Hospital of Kunming Medical University, Kunming Medical University, Kunming, China; ^2^Department of Gastrointestinal and Hernia Surgery, First Affiliated Hospital of Kunming Medical University, Kunming, China; ^3^Scientific Research Laboratory Center, First Affiliated Hospital of Kunming Medical University, Kunming, China; ^4^Department of Dermatology, Second People’s Hospital of Dali City, Dali, China

**Keywords:** HIV/AIDS, gut microbiota, immune reconstitution, probiotics, fecal bacteria transplantation

## Abstract

Human immunodeficiency virus type 1 (HIV-1) infection of CD4^+^ T cells in the gut plays an insidious role in acquired immunodeficiency syndrome (AIDS) pathogenesis. Host immune function is closely related to gut microbiota. Changes in the gut microbiota cause a different immune response. Previous studies revealed that HIV-1 infection caused changes in gut microbiota, which induced immune deficiency. HIV-1 infection results in an abnormal composition and function of the gut microbiota, which may disrupt the intestinal epithelial barrier and microbial translocation, leading to long-term immune activation, including inflammation and metabolic disorders. At the same time, an abnormal gut microbiota also hinders the effect of antiviral therapy and affects the immune reconstruction of patients. However, studies on the impact of the gut microbiota on immune reconstitution in patients with HIV/AIDS are still limited. In this review, we focus on changes in the gut microbiota caused by HIV infection, as well as the impact and regulation of the gut microbiota on immune function and immune reconstitution, while we also discuss the potential impact of probiotics/prebiotics and fecal microbiota transplantation (FMT) on immune reconstitution.

## Introduction

With the development of antiretroviral therapy (ART), the mortality and morbidity associated with HIV/AIDS have decreased. ART can effectively inhibit viral replication, and it generally improves immune status to achieve immune reconstitution. However, there are still some patients with AIDS who are called immune non-responders (INRs) who cannot achieve immune reconstitution.

In humans, more than 80% of lymphocytes are located in the intestinal mucosa, and approximately 60% of CD4^+^ T lymphocytes are located in intestinal-associated lymphoid tissues. Hence, the intestinal mucosal immune system is one of the main targets of HIV attack, which decreases the intestinal mucosal barrier function and increases bacterial translocation (Brenchley and Douek, 2008). An increasing number of studies have demonstrated that the gut microbiota composition and function change in AIDS, but the change cannot be completely recovered after ART (Cohen, 2005; Moeller et al., 2013; Zilberman-Schapira et al., 2016). Therefore, changes in the intestinal microbiota may affect the recovery of immune function. The potential mechanism includes the formation of a virus shelter, resistance to ART, promotion of intestinal mucosal barrier damage, and further entry of intestinal bacteria, and their metabolites into the circulatory system, resulting in long-term immune activation, inflammation, and metabolic disorder (Zilberman-Schapira et al., 2016). These results indicate that the gut microbiota plays an essential role in the reconstitution of immune function in patients with AIDS. Consequently, the gut microbiota has become a major point of focus in HIV research.

**Figure 1:**
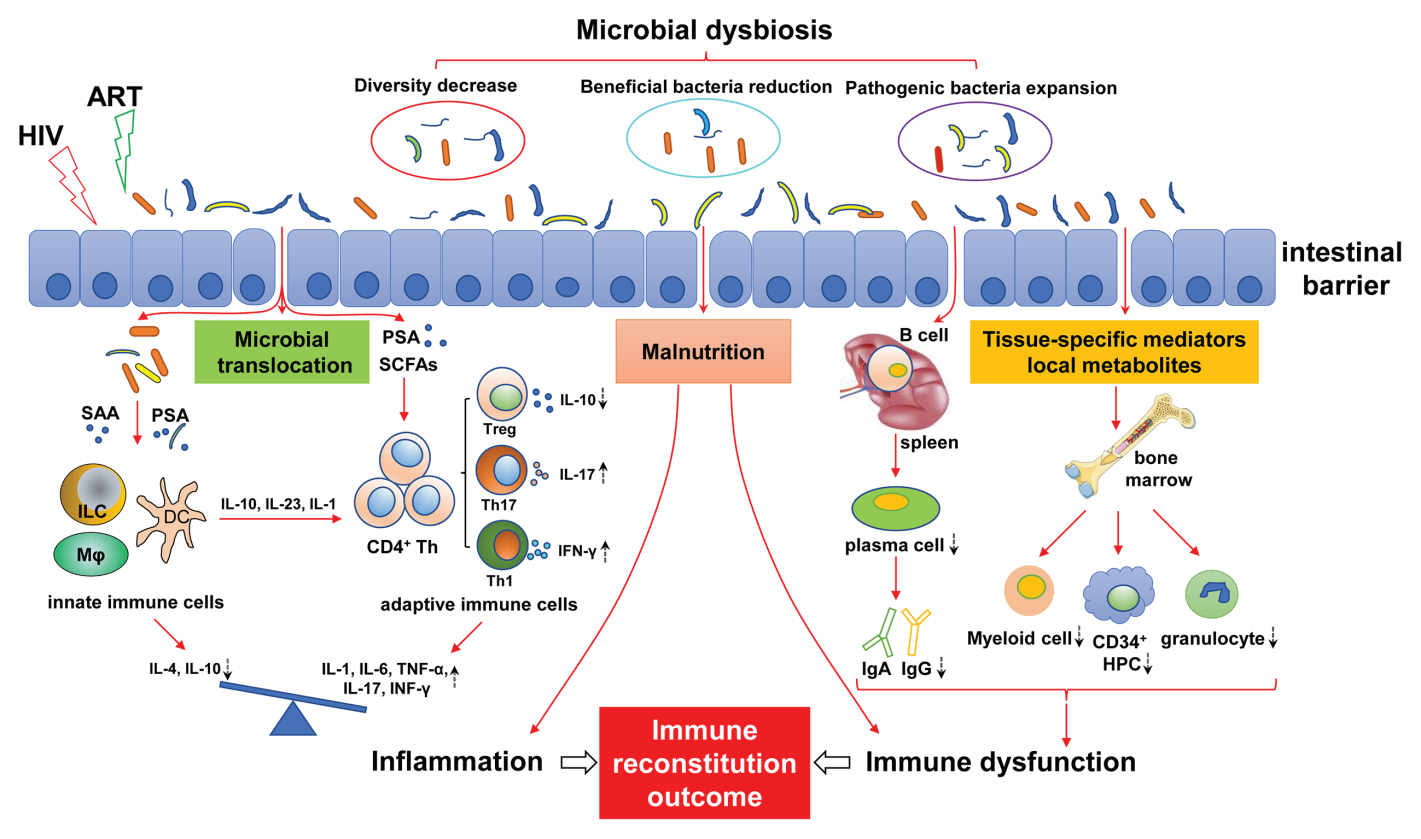
Mechanisms of gut microbial dysregulation affecting immune reconstitution. HIV infection leads to intestinal microbial dysregulation, including decreased abundance, reduced beneficial flora, and increased pathogenic bacteria. Intestinal barrier injury further aggravates the imbalance of gut microbiota, causing the translocation of gut microbiota and its metabolites (e.g., SAA, PSA, and SCFAs), resulting in continuous innate and adaptive immune activation. The immune response of the activated immune system is disordered and is characterized by a pro-inflammatory immune response. The imbalance of differentiation between innate immune cells and adaptive immune cells (e.g., Treg/Th17) leads to an increase in pro-inflammatory cytokines (IL-1, IL-6, TNF-α, and IFN-γ) and a decrease in anti-inflammatory cytokines (IL-4 and IL-10), resulting in chronic inflammation. Dysfunctional intestinal microbiota can affect the development of bone marrow cells by regulating local metabolites and tissue-specific mediators, resulting in a decrease in myeloid cells, CD34^+^ hematopoietic progenitor cells (HPC), and granulocytes. Similarly, intestinal flora affect the differentiation of B cells into plasma cells in the spleen, resulting in abnormal antibody differentiation, such as decreased IgA and IgG. In addition, malnutrition caused by HIV infection and intestinal flora disorder can also lead to immune dysfunction, inflammatory response, bone marrow hematopoietic cell dysplasia, and antibody production abnormalities. Antiretroviral therapy (ART) may exacerbate this effect. The dashed arrows indicate an increase or decrease in cells or cytokines. ART, antiviral therapy; SAA, serum amyloid A; PSA, polysaccharide A; SCFAs, short-chain fatty acids; ILC, innate lymphocytes; HPC, hematopoietic progenitor cell.

Here, we review the role of the gut microbiota in immunomodulation, and we focus on the changes in gut microbiota in people infected with HIV and the effects on immune reconstitution (Figure 1) and explore novel, effective, specific therapeutic strategies to restore gut microbiota composition and function, thus helping to improve immune reconstitution.

## Human Gut Microbiota

The human gut microbiota (HGM) is a vast microbial community that is crucial to human health and consists of different types of microbes, including bacteria, archaea, eukaryotes, viruses, and parasites (Lozupone et al., 2012). The gastrointestinal microenvironment mainly favors the growth of seven types of bacteria, including *Firmicutes*, *Bacteroidetes*, *Actinobacteria*, *Fusobacteria*, *Proteobacteria*, *Verrucomicrobia*, and *Cyanobacteria* (Backhed et al., 2005). Among them, *Bacteroides* and *Firmicutes* dominate more than 90% of the total bacteria. Different parts of the gastrointestinal tract have specific flora compositions due to their different microenvironments, and the number and diversity of microorganisms grow vertically (Eckburg et al., 2005; Donaldson et al., 2016).

There are substantial individual differences in the gut microbiota. According to the report of the Human Microbiome Project Consortium in 2012, there are dramatic differences in the composition and richness of gut microbiota in healthy people (Human Microbiome Project Consortium, 2012). Gut microbes are influenced by multiple factors, including mode of birth, sex, geographic location, diet, age, medications, and stress (Cresci and Bawden, 2015), which shape the critical role of intestinal microbes in health and disease. Increasing evidence has showed that gut microbiota is involved in several physiological functions of the host, promotes growth and development, and maintains normal physiological activities, including digestion, metabolism, nutrient absorption, regulation of immune function, and vitamin synthesis (Table 1; Adak and Khan, 2019). However, dysbioses of the gut microbiota is associated with unhealthy and disordered treatment outcomes (Marchesi et al., 2016; DeJong et al., 2020; Vieira-Silva et al., 2020).

**Table 1:** Functions of gut microbiota.

Function type	Specific performance
Neurological	Control the ENS as well as CNS: production, expression, and turnover of neurotransmitters and neurotrophic factors; maintain intestinal barrier and tight junction integrity; modulate the enteric sensory afferents; produced bacterial metabolites mucosal immune regulation
Metabolic	Dietary fibers fermentation; short chain fatty acids (SCFAs) production; protein and amino acid metabolism; bile salt biotransformation
Structural	Tight junction regulation; mucus layer properties; crypt and villi development; villi microvascularization
Protective	Improve nutrition; maintain intestinal barrier; immune activation; antimicrobial peptides (AMPs) secretion

## HIV Infection Changes Host Microbial Diversity

In patients with HIV, including those who are receiving ART to control the disease, the composition of the gut microbiota is significantly different from that of healthy people. For these patients, gut microbial imbalance may lead to the collapse of intestinal immune function, causing systemic bacterial proliferation and inflammation. According to previous animal experiments and clinical data, the gut microbial imbalance of people infected with HIV mainly includes changes in microbial diversity, decreases in symbiotic beneficial bacteria, and increases in potentially pathogenic bacteria (Dillon et al., 2016a; Deusch et al., 2018).

With the development of microbial sequencing technology, it has been shown that the gut microbial diversity of people infected with HIV is significantly different between 16S sRNA gene sequencing and next-generation sequencing (NGS). Since the changes in gut microbiota during HIV infection are affected by complex factors such as population, age, sex, duration of infection, sample type, and ART (Li et al., 2016), the changes in gut microbiota diversity observed in previous studies are not always consistent. Multiple studies have shown that the α-diversity of the intestinal microbiota in people infected with HIV is decreased (McHardy et al., 2013; Mutlu et al., 2014; Nowak et al., 2015; Vazquez-Castellanos et al., 2015; Dubourg et al., 2016; Monaco et al., 2016; Sun et al., 2016; Zhou et al., 2018; Lu et al., 2019). Other studies found that the α-diversity index increased significantly between healthy controls and patients infected with HIV (Lozupone et al., 2013) or remained unchanged (Dillon et al., 2014; Dinh et al., 2015; Ling et al., 2016). A meta-analysis of 1,032 samples (311 HIV^−^ and 721 HIV^+^) involving 17 studies (Tuddenham et al., 2020) showed that HIV infection is associated with decreased intestinal microbial α-diversity. However, the further stratified analysis found that there was no significant correlation between HIV^+^ status and α-diversity among men who having sex with men (MSM), while the α-diversity of HIV^+^ patients decreased significantly when it was limited to women and MSM. The results showed that sex and sex risk categories affected α-diversity. Therefore, future research should consider these factors to determine the relationship between gut microbiota and HIV more accurately. In addition, it should be noted that the diversity of gut microbiota in patients with ART cannot return to the state before infection (Lozupone et al., 2013; Nowak et al., 2015). Despite the differences among different studies in the changes in microbial diversity, changes in intestinal microbial diversity are related to lower CD4^+^ T cell counts, higher inflammation, and increased immune activation in people infected with HIV (Dillon et al., 2016a; Serrano-Villar et al., 2017a; Annavajhala et al., 2020).

The change in gut microbial diversity will not only affect disease progression but also lead to changes in immune status. The changes in α-diversity were correlated with inflammatory/microbial translocation and negatively correlated with circulating sCD14, LPS, LBP, and sCD163 (Nowak et al., 2015), and patients with lower levels of CD4^+^ T cells had less diversity and abundance of gut microbiota (Monaco et al., 2016). Also confirmed that the low T cell count in peripheral blood was correlated with a decrease in intestinal bacterial diversity and richness and an increase in the abundance of specific bacteria, such as *Enterobacteriaceae*, which was linked to inflammation (Monaco et al. 2016). There were significant differences in the intestinal microbial diversity between HIV positive individuals and healthy individuals, which were positively correlated with C-reactive protein (CRP) levels and T cell activation in HIV^+^ individuals (Vazquez-Castellanos et al., 2015). This further indicated that the loss of intestinal microbial diversity was related to the impairment of immune function, bacterial translocation, and systemic inflammation. Furthermore, low microbial diversity and dysfunctional bacterial metabolic pathways exacerbate systemic inflammation in HIV infection (El-Far and Tremblay, 2018). This main reason is that HIV leads to the disruption of the intestinal epithelial barrier, resulting in the loss of tight junctions and apoptosis of intestinal cells, which gives rise to the increased translocation of microorganisms and microbial products, and HIV-mediated depletion of CD4^+^ Th17 cells and increased cytokine expression can induce sustained activation of immune cells and the production of high levels of inflammatory cytokines in circulation, such as IL-1β, IL-6, and TNF-α (Tincati et al., 2016). In addition, several studies have linked viral load, the CD4/CD8 ratio, system biomarkers, cytokines, immune activation, bacterial translocation, and thymus function with intestinal microbial diversity (Perez-Santiago et al., 2013; Dillon et al., 2014; Mutlu et al., 2014; Nowak et al., 2015). This indicates that the change in intestinal microbial diversity plays an important role in the progression of HIV and immune deficiency.

## HIV Infection Changes the Host Microbial Composition

The gut microbiota includes beneficial, opportunistic, and harmful bacteria in healthy people. In addition to the change in intestinal microbial diversity, HIV also changes its composition, that is, beneficial symbiotic bacteria decrease and harmful pathogens increase, mainly the accumulation of inflammatory Gram-negative bacteria and potential pathogens. However, the characteristic changes in the microbial community associated with HIV infection were only obvious in chronically infected people but not in patients with acute HIV infection (Rocafort et al., 2019). Recent studies have shown that the average counts of beneficial bacteria, such as *Bacteroides*, *Faecalis*, *Bacteroides vulvae*, *Diplococcus*, and *Arbuscular Roseus*, were lower compared with healthy controls, but a higher proportion of potentially pathogenic microorganisms, including *Proteus*, *Enterococcus*, *Klebsiella*, and *Streptococcus*, and natural microorganisms, including *Lactobacillus* and *Lactococcus*, is present (Zhou et al., 2018). These beneficial bacteria may interact with gut-associated lymphoid tissue (GALT) to maintain intestinal integrity, thereby reducing the possible transport of microbial products, and pathogens may be related to the progression of AIDS, immune activation, and chronic inflammation. Moreover, it has been reported that the changes in intestinal microbiota in patients with HIV infection mainly include *Proteobacteria*, *Bacteroides*, and *Firmicutes bacteria*, in which *Proteobacteria* and *Firmicutes* increased significantly while *Bacteroides* decreased significantly (Dillon et al., 2014, 2016a, Sun et al., 2016, Zhou et al., 2018). These changes will eventually lead to microbial imbalance and destruction of the intestinal mucosal epithelial barrier, aggravating microbial translocation, inflammation, and immune activation (Zevin et al., 2016).

Considering the role of the intestinal microbiota in immunity, the reduction in beneficial symbiotic bacteria and the increase in harmful pathogens will undoubtedly cause immune deficiency and inflammation. HIV infection is accompanied by an increase in pro-inflammatory or potentially pathogenic bacterial populations (e.g., *Pseudomonas aeruginosa* and *Candida albicans*), as well as a decrease in the level of beneficial bacteria, such as *Bifidobacterium* and *Lactobacillus*, which are associated with the impairment and loss of mucosal barrier function (Perez-Santiago et al., 2013; Dillon et al., 2014), whereas the damage and loss of mucosal barrier function were associated with immune status (Nwosu et al., 2014; Nowak et al., 2015). A case-control study from France (Dubourg et al., 2016) showed that the abundance of *Faecalibacterium prausnitzii* was decreased, which was negatively correlated with inflammation/immune activation, while the abundance of *Enterobacteriaceae* and *Enterococcaceae* positively correlated with inflammation/immune activation, which was higher in HIV infection. The number of beneficial bacteria, such as *Bifidobacterium* and *Lactobacillus*, in patients with AIDS is significantly lower than that in healthy people, and the numbers of *Escherichia coli, Enterococcus faecalis*, and *Enterococcus faecium* increase; and this change is related to the levels of TNF-α and CD4^+^ T lymphocytes in circulation (Lu et al., 2019). Composition changes in the gut microbiota will destroy gut homeostasis in patients with HIV infection to induce systemic immune activation, thus further damaging the intestinal barrier function, increasing bacterial translocation and increasing systemic inflammation, which leads to the progression of AIDS (Marchetti et al., 2013; Assimakopoulos et al., 2014).

Furthermore, it has been shown that, through the comparison of different populations, the composition of gut microbiota in people infected with HIV has a common pattern in which the enrichment of *Erysipelotrichaceae*, *Enterobacteriaceae*, *Desulfovibrionaceae*, and *Fusobacteria* and the depletion of *Lachnospiraceae*, *Ruminococceae*, *Bacteroides*, and *Rikenellaceae* are associated with inflammation and AIDS progression (Lozupone et al., 2013). Multiple cross-sectional studies show that the intestinal microbiota changes from *Bacteroides* to *Prevotella* after HIV infection (Mutlu et al., 2014; Vazquez-Castellanos et al., 2015; Dillon et al., 2017; Serrano-Villar et al., 2017b; Vujkovic-Cvijin and Somsouk, 2019), and the genera *Phascolarctobacterium*, *Megamonas*, *Dialister*, and *Clostridium* XIVb persisted, which are significantly associated with systemic inflammatory cytokines; this change has not been fully reversed even after ART (Ling et al., 2016).

In addition to the changes in gut microbiota caused by HIV, ART may also affect the composition and function of gut microbiota. Studies have shown that the initiation of ART is related to the relative increase in *Fusobacterium*, *Proteobacteria*, and *Tenella* and the decrease in *Bacteroides* and *Firmicutes* (Nowak et al., 2015; Pinto-Cardoso et al., 2017; Villanueva-Millan et al., 2017). At the same time, there is evidence that ART can induce changes in the microbiota independent of HIV infection, suggesting that ART may aggravate the imbalance of intestinal microbiota (Lozupone et al., 2014; Nowak et al., 2015; Noguera-Julian et al., 2016). A recent study (Villanueva-Millan et al., 2017) evaluated the effects of ART on intestinal microbial composition and microbial translocation. It turns out that there was a significant loss of intestinal microbial diversity and an increase in imbalance after ART, while this phenomenon was not observed in another study (Nowak et al., 2015). The heterogeneity of the ART protocol and immunovirological status among different studies may lead to different results.

## Effects of Gut Microbiota Changes on Immune Reconstitution of Individuals Infected With HIV

Antiretroviral therapy can effectively inhibit HIV replication. Most patients can achieve significant viral load reduction and peripheral blood CD4^+^ T cell reconstruction. However, although some patients achieve virological suppression after ART, the reconstruction of CD4^+^ T cells is still insufficient (Lu et al., 2015); these individuals are known as INRs, and the prevalence ranges from 10 to 40% (Massanella et al., 2013; Nakanjako et al., 2016). Compared with those who had good immune reconstitution, the INRs showed chronic immune activation and high inflammatory state, which was related to the high incidence rate of cardiovascular diseases, metabolic diseases, and cancer (Paiardini and Muller-Trutwin, 2013; Nix and Tien, 2014).

Among the factors affecting immune reconstitution, changes in the gut microbiota are one of the key factors (Yang et al., 2020). Dysregulation of the gut microbiome has been associated with the recovery of CD4^+^ T cells (Lu et al., 2018), which is the main determinant of immune reconstitution after cART (Kaufmann et al., 2005; Brenchley et al., 2006b; Marchetti et al., 2008; Lu et al., 2018). Recent data suggest that the decrease in CD4^+^ T cell count in peripheral blood is related to microbial translocation and an increase in *Enterobacteriaceae* (Moore and Keruly, 2007; Monaco et al., 2016). However, ART can only partially restore gastrointestinal function damage and gut microbiota, and the recovery of intestinal homeostasis is still hindered. Although the microbial metabolites (such as circulating LPS and bacterial DNA) in the circulation are less than those of untreated patients, they are still higher than the normal level, suggesting that microbial translocation still exists, which affects the recovery of immune function (Brenchley et al., 2006a,b; Jiang et al., 2009; Hooper and Macpherson, 2010; Ponte et al., 2016; Lu et al., 2018; Zhou et al., 2018). Serrano-Villar et al. also demonstrated that intestinal microbes affect the full recovery of CD4^+^ T cells in patients with HIV infection receiving ART (Serrano-Villar et al., 2016). However, how the imbalance of intestinal microbiota leads to the failure of immune reconstitution is still unclear.

Immune non-responders show severe immune dysfunction, and the influencing factors include bone marrow hematopoietic progenitor cell reduction, thymus dysfunction, abnormal immune activation, immune failure, imbalance of immune regulatory cells, such as regulatory T cells (Tregs) and Th17 cells, and continuous replication of the virus in reservoirs (Yang et al., 2020). Considering the close relationship between the gut microbiota and the immune system, gut microbiota dysbiosis may mediate immune dysfunction of INRs, thus affecting immune reconstitution (Figure 1).

Alterations in intestinal microbes can affect the development, differentiation, and maturation of immune cells. It is known that immune cells are mainly produced by bone marrow hematopoietic stem cells. However, the absence of a gut microbiota leads to decreased myeloid cells in the bone marrow and results in delayed clearance of systemic bacterial infections (Marchetti et al., 2008). This indicates that the gut microbiome influences the development of bone marrow hematopoietic cells. During the various phases of bone marrow cell development, the gut microbiota is engaged at every stage, which affects the migration and gene expression of tissue-resident myeloid cells and the production of bone marrow and circulating granulocytes by regulating local metabolites and tissue-specific mediators (Serrano-Villar et al., 2016). Similarly, the gut microbiota also regulates the maturation of circulating myeloid cells, such as neutrophils and basophils, by driving toll-like receptor (TLR)- and myeloid differentiation factor 88 (MyD88)-mediated signaling pathways (Khosravi et al., 2014; Thaiss et al., 2016). In addition to its effect on the development of the myeloid arm in the congenital immune system, the gut microbiota is implicated in regulating innate lymphocytes (ILCs). ILCs are a group of innate immune cells that are composed of cytotoxic cells (NK cells) and non-cytotoxic subpopulations (ILC1, ILC2, and ILC3; Zhang et al., 2015). Although there is still a contradiction regarding whether the development of ILCs requires the participation of gut microbiota (Kiss et al., 2011; Hill et al., 2012), gut microbiological signals certainly affect the maturation and acquisition of normal ILC function (Sawa et al., 2010). Intestinal bacteria can directly signal through the pattern recognition receptor (PRR) on ILC3s or indirectly regulate intestinal myeloid cells and epithelial cells to affect the function of ILC3s (Sonnenberg and Artis, 2012).

Like the innate immune system, the gut microbiota also influences adaptive immunity. Severe intestinal immune deficiency was found in GF animals, including impaired development of GALTs, gut-associated Th17 cells, and Tregs, decreased IgA-producing B cells, and intra-epithelial CD8^+^ T cells. This suggests that the gastrointestinal immune system relies heavily on gut microbiota. Although the mechanism of gut microbiota regulating adaptive immunity is still unknown, it is clear that some gut microbiota can affect the differentiation of immune cells through a variety of different mechanisms (Sawa et al., 2011). For example, it has been confirmed that *segmented filamentous bacteria* (SFB) can strongly induce the differentiation of Th17 cells by upregulating serum amyloid A (SAA) expression to stimulate CD11c^+^ DCs to produce IL-6 and IL-23 (Francino, 2014; Pickard et al., 2017). *Clostridium* IV and XIVa can stimulate colon epithelial cells to produce TGF-β, which can induce T cells to differentiate into Foxp3^+^ Tregs (Gaboriau-Routhiau et al., 2009). At the same time, *Clostridium* is a major producer of short-chain fatty acids (SCFAs), such as butyric acid, propionic acid, and acetic acid, which mediate immune regulation by inducing CD4^+^ T cells to differentiate into Tregs (Ivanov et al., 2009; Atarashi et al., 2011; Smith et al., 2013). *Bacteroides fragilis* can produce polysaccharide A (PSA) and regulate the transformation of CD4^+^ T cells to Foxp3^+^ Tregs in a TLR2-dependent manner (Arpaia et al., 2013; Furusawa et al., 2013). Tregs can secrete IL-10 to regulate the response of Th17 and Th1 (Round and Mazmanian, 2010) and regulate the homeostasis of iNKT cells (Mazmanian et al., 2008). In addition, the microbiota is crucial for the maturation, differentiation, and antibody production of B cells. Studies have shown that microbial antigens and microbial metabolites, such as SCFAs, strongly promote plasma cell differentiation in mucous membranes and the whole body (Mazmanian et al., 2005). The number of IgA^+^ plasma cells and the abundance of IgA in the intestine decreased when intestinal microbial stimulation was lacking (An et al., 2014; Kim et al., 2016). In addition to IgA, the production of IgM, IgD, IgE, and IgG seems to be related to gut microbiota (Hapfelmeier et al., 2010; Lecuyer et al., 2014; Koch et al., 2016), but how intestinal microbes affect the production of these antibodies needs to be further explored.

Gut microbiota dysbiosis may hinder immune reconstitution by driving immune activation and chronic inflammation, which cannot be completely controlled by ART and is related to the low efficiency of CD4^+^ T cell reconstruction (Kaufmann et al., 2005; Brenchley et al., 2006b; Marchetti et al., 2008; Lu et al., 2018). HIV infection induces a reduction in the number of bacteria that are important in maintaining the health of the epithelial barrier and immune regulation while increasing the number of bacteria with pro-inflammatory potential, which is related to immune status and affects immune reconstruction (McHardy et al., 2013; Mutlu et al., 2014; Vazquez-Castellanos et al., 2015; Choi et al., 2017). Compared with healthy controls, the relative abundance of *Prevotella* in individuals infected with HIV increased significantly, while the *Bacteroides* abundance decreased (Mutlu et al., 2014; Vazquez-Castellanos et al., 2015; Dillon et al., 2017; Serrano-Villar et al., 2017b; Vujkovic-Cvijin and Somsouk, 2019). The relative abundance of *Prevotella* was positively correlated with the activation of many different immune cells, including intestinal mucosal T cells and DCs (Dillon et al., 2014), whereas DC activation in mucosal tissues was associated with increased mucosal viral load, mucosal and systemic T cell activation, and plasma and mucosal cytokine production (Pickard et al., 2017). Meanwhile, the depletion of *Bacteroides* (including *B. fragilis*) is associated with systemic immune activation and chronic inflammation (Choi et al., 2017). Additionally, the abundance of *Clostridium*, *Enterococcus faecalis*, and other potential pathogens in INRs was higher than that in healthy controls, and the abundance of these bacteria was negatively correlated with CD4^+^ T cell count (Vujkovic-Cvijin et al., 2013; Dillon et al., 2016b). Pérez-Santiago et al. (Lee et al., 2018) found that *Lactobacilli* decreased in patients infected with HIV, while *Lactobacilli* could regulate the anti-inflammatory immune response and participate in maintaining the integrity of the intestinal mucosal barrier, which was associated with increased CD4 cell count, decreased microbial translocation, and decreased systemic immune activation. These observations suggest that altered gut microbiota may be associated with abnormal immune activation and chronic inflammation, thus contributing to poor immune reconstitution in individuals infected with HIV-1.

Alterations in gut microbes disrupt gut homeostasis, and there is evidence that dysbiosis of the gut microbiota is the cause of immune response disorder and intestinal barrier disruption. The intestinal barrier is destroyed in the early stage of HIV infection, which is associated with gut microbiota dysbiosis (Perez-Santiago et al., 2013). The disruption of intestinal barrier integrity seriously affects the absorption of nutrients, thus hindering the recovery of immune function. Recently, several cross-sectional studies (Mudd and Brenchley, 2016; Gebremichael et al., 2018; Daka and Ergiba, 2020) have shown that the prevalence of malnutrition in patients with HIV/AIDS treated with ART is over 23%, and severe malnutrition amounted to 9%. Further analysis revealed that CD4^+^ T cell counts below 350 or 200 cells/μl were significantly associated with malnutrition. In the case of malnutrition, changes in the levels of immune cell populations, hormones, and cytokines lead to changes in the metabolism of immune cells, thereby affecting immune function (Oumer et al., 2019). During the whole process of HIV infection, nutritional status and immune function are closely related and interact. Malnutrition constantly damages the immune system and is associated with immunosuppression (Ivanov et al., 2009), which may be one of the reasons for poor immune function reconstruction. The close interaction between gut microbiota and food intake not only helps to degrade food nutrients but can also synthesize a variety of nutrients for human use, which is regarded as a key aspect of nutrition (Zilberman-Schapira et al., 2016; Alwarawrah et al., 2018; Valdes et al., 2018). Alterations in gut microbiota in individuals infected with HIV may lead to malnutrition and, thus, hinder immune reconstitution.

In addition, changes in intestinal microbes resulting from HIV infection may help HIV escape immune surveillance, establish effective and persistent infections in cells or tissues, and establish viral shelters that are resistant to ART (Zilberman-Schapira et al., 2016), which are known as HIV latent reservoirs. Viruses in latent HIV reservoirs can hide in the cell and assume a dormant state, escape the attack of cellular immunity and humoral immunity, and survive for a long time, thus forming a persistent infection. This may also be one of the factors affecting immune reconstitution after ART. Studies have shown that the host recognizes invading viruses through a variety of mechanisms (Kumar et al., 2011; Broz and Monack, 2013; Kieser and Kagan, 2017), while viruses achieve immune escape by targeting immune sensors, adapter molecules, intracellular kinases, and transcription factors [e.g., TLRs, NOD-like receptor (NLR), retinoic acid-inducible gene I (RIG-I), IFN-β promoter stimulator-1, and IFN gene axis] for persistent infection (Abe et al., 2019). Although the mechanism of HIV immune escape is currently unknown, considering that changes in intestinal microbes caused by HIV are associated with immune activation, intestinal microbial dysregulation may be beneficial to HIV immune escape, and intestinal microbial-mediated immune escape may be one of the reasons for poor immune reconstitution. HIV infection is associated with a marked inflammatory response and immune activation that does not disappear completely with ART. Given the close link between intestinal microbes and immunity, intestinal microbes caused by HIV infection play an important role in this change (Estrada and Gonzalez, 2018). Increased levels of pro-inflammatory and potentially pathogenic bacteria in the HIV-infected population contribute to increased levels of inflammatory cytokines in the intestine or circulation, including TNF-α, IL-6, IL-1β, IFN-γ, IL-17, and IL-22, resulting in the formation of an inflammatory environment. HIV damage to the intestinal barrier exacerbates the translocation of intestinal bacteria, resulting in systemic inflammation (Vazquez-Castellanos et al., 2015). In addition, studies have reported a significant increase in Tregs in individuals infected with HIV (Bi et al., 2009; Suchard et al., 2010) and disruption of the Treg/Th17 balance *in vivo*, thus forming an immunosuppressive microenvironment, while intestinal microbes play an important role in the induction and regulation of Tregs (Cheng et al., 2019). Thus, dysregulated intestinal microbes may lead to HIV evasion from the attack of the epidemic system by mediating the formation of inflammatory and immunosuppressive environments, similar to immune escape of tumors. Of course, the impact and mechanism of intestinal microbial dysbiosis on HIV immune escape and the impact of immune escape on immune reconstitution remain to be elucidated.

## Interventions to Regulate Gut Microbiota

Because various changes in intestinal microbes during HIV infection are closely related to immune function, ART cannot fully restore the resulting local and systemic inflammation and loss of CD4^+^ T cells, chronic immune activation, and immune dysregulation. Developing strategies to help restore intestinal microecology is beneficial for immune reconstitution in patients with HIV-infection, especially those with poor immune reconstitution after ART.

Probiotics are living microorganisms that are beneficial to the health of the host (Hill et al., 2014), and prebiotics are a kind of healthy substrate selectively utilized by host microorganisms, which are widely used because they can benefit the treatment and prevention of diseases by altering the microbiota and/or regulating their functional equivalents (Sanders et al., 2019). Whether the intervention of probiotics and prebiotics can be used to help immune reconstitution has become a research hotspot in the treatment of patients with HIV-infection.

Normal microbiota have a tremendous impact on metabolism and inflammation. HIV infection imbalances intestinal microbes and affects the differentiation of CD4^+^ Th17 cells, and the balance between CD4^+^ Th17/Treg lymphocytes is disrupted. Probiotics can upregulate Treg activation and inhibit pro-inflammatory immune responses, which provides a basis for probiotics in combination with ART in HIV-1 (Cunningham-Rundles et al., 2011). Current evidence suggests that probiotics/prebiotics can improve microbial translocation, regulate intestinal microbes, promote CD4^+^ T cell reconstitution, and reduce the activity of indoleamine 2,3-dioxygenase-1 (IDO-1), contributing to the protection of Th17 cells during infection (Vujkovic-Cvijin et al., 2015; Ortiz et al., 2016). Taking probiotics or prebiotics can increase CD4^+^ T cell counts in peripheral blood, reduce immune activation, alter intestinal flora, and reduce sCD14 and bacterial DNA concentrations in plasma (Trois et al., 2008; Gori et al., 2011; Hummelen et al., 2011; Gonzalez-Hernandez et al., 2012; Hemsworth et al., 2012). D’Ettorre et al. (2017) found that in HIV^+^ individuals treated with ART, supplementation with probiotics promoted a decrease in T cell activation and an increase in Th17 frequency, and probiotics were also associated with restoration of intestinal epithelial barrier integrity, reduction in intra-epithelial lymphocyte density and intestinal cell apoptosis, and improvement in mitochondrial morphology caused in part by heat shock protein 60 (Hsp60) regulation. It can be seen that probiotic supplements have potential benefits for the physical and immune barrier integrity reconstruction of the intestinal mucosa of HIV-positive patients receiving ART, which is conducive to immune reconstitution. Furthermore, in patients with HIV-infection receiving ART, supplementation with probiotics increases T lymphocytes, significantly reduces the levels of TGF-β, IL-10, IL-12, and IL-1β, and has immunological and virological benefits (Falasca et al., 2015; Ishizaki et al., 2017). These results suggest that probiotics given to HIV-positive individuals often have positive outcomes.

Current studies all support that probiotics/prebiotics can be beneficial for the treatment of HIV infection, and no serious adverse reactions have been identified in the assessment of the risk of probiotic use (Carter et al., 2016). Only one case of *Lactobacillus acidophilus bacteremia* associated with AIDS linked to excessive consumption of probiotic yogurt has been reported (Haghighat and Crum-Cianflone, 2016). This suggests that probiotic supplementation is a safe and effective measure to regulate intestinal microorganisms.

Fecal microbiota transplantation (FMT) is the transfer of feces from healthy donors into the colon of patients who become ill as a result of microbiome changes, intending to normalize the composition and function of the intestinal microbiota, thereby curing the disease (Khoruts and Sadowsky, 2016; Vindigni and Surawicz, 2017). This may be a new way to restore the intestinal microbiota of people infected with HIV. Elopre et al. first reported the successful use of FMT in the treatment of recurrent refractory *Clostridium* infection in patients with HIV-infection (Elopre and Rodriguez, 2013). In immunocompromised patients, including those with HIV infection, FMT is an effective treatment for refractory *Clostridium difficile* recurrent infections with a similar incidence of serious adverse events as in immunocompromised patients (Di Bella et al., 2015; Shogbesan et al., 2018). A recent study conducted a pre-clinical evaluation of the safety and efficacy of FMT as a potential treatment for HIV-infected patients and found that the number of Th17 and Th22 cells in peripheral blood increased significantly after FMT, and the activity of CD4^+^ T cells in gastrointestinal tissues decreased. Importantly, the transplantation was well tolerated without any adverse clinical side effects (Hensley-McBain et al., 2016). Although HIV-infected patients tolerate FMT well, only part of the donor microbiome was implanted, and systemic inflammation markers did not change significantly after FMT (Vujkovic-Cvijin et al., 2017). In addition, considering the potential risk of serious adverse events likely due to transmission of pathogenic organisms (Azimirad et al., 2019), the clinical practice of FMT should fall under the regulations of investigative new drugs (INDs) recommended by the United States Food and Drug Administration (FDA; Moore et al., 2014; Kelly and Tebas, 2018). Therefore, the feasibility and effectiveness of FMT for patients with HIV-infection call for further studies and comprehensive evaluations.

## Conclusion

Current research has demonstrated that HIV infection causes changes in intestinal microbial diversity and the specific bacterial composition. Nevertheless, the regulatory effect of gut microbiota on immune function is well known. The diversity and composition of the gut microbiota change in infected individuals with poor immune recovery, which may be the key factor for poor immune reconstitution in some infected individuals. Probiotics/prebiotics and FMT can restore the diversity of intestinal microorganisms to a certain extent, repair the intestinal barrier, alleviate the inflammatory response, and promote the reconstruction of immune function. However, it is not effective for all patients and there are some limitations. Although significant progress has been made in the study of gut microbiota, it is still necessary to determine whether the changes in gut microbiota mediate the pathological changes and poor immune reconstitution of people infected with HIV to provide further theory and direction for the treatment strategy of microorganisms for immune reconstitution.

## Author Contributions

S-TG, Y-QK, and K-HW conceived and designed the paper. S-TG undertook all reviews and extracted the data, which was verified by Y-QK. S-TG, Z-YZ, Y-XW, DL, JY, J-BZ, Y-QK, and K-HW interpreted the data. S-TG drafted the manuscript, and Y-QK and K-HW critically revised it. All authors listed have made a substantial, direct, and intellectual contribution to the work, and all read and approved the final manuscript.

### Conflict of Interest

The authors declare that the research was conducted in the absence of any commercial or financial relationships that could be construed as a potential conflict of interest.
